# Rectal Application of a Highly Osmolar Personal Lubricant in a Macaque Model Induces Acute Cytotoxicity but Does Not Increase Risk of SHIV Infection

**DOI:** 10.1371/journal.pone.0120021

**Published:** 2015-04-08

**Authors:** Sundaram A. Vishwanathan, Monica R. Morris, Richard J. Wolitski, Wei Luo, Charles E. Rose, Dianna M. Blau, Theodros Tsegaye, Sherif R. Zaki, David A. Garber, Leecresia T. Jenkins, Tara C. Henning, Dorothy L. Patton, R. Michael Hendry, Janet M. McNicholl, Ellen N. Kersh

**Affiliations:** 1 National Center for HIV/AIDS, Viral Hepatitis, STD and TB Prevention, Atlanta, Georgia, United States of America; 2 National Center for Emerging & Zoonotic Infectious Diseases, Centers for Disease Control and Prevention, Atlanta, Georgia, United States of America; 3 University of Washington, Seattle, Washington, United States of America; University of Pittsburgh, UNITED STATES

## Abstract

**Background:**

Personal lubricant use is common during anal intercourse. Some water-based products with high osmolality and low pH can damage genital and rectal tissues, and the polymer polyquaternium 15 (PQ15) can enhance HIV replication *in vitro*. This has raised concerns that lubricants with such properties may increase STD/HIV infection risk, although *in vivo* evidence is scarce. We use a macaque model to evaluate rectal cytotoxicity and SHIV infection risk after use of a highly osmolar (>8,000 mOsm/kg) water-based lubricant with pH of 4.4, and containing PQ15.

**Methods:**

Cytotoxicity was documented by measuring inflammatory cytokines and epithelial tissue sloughing during six weeks of repeated, non-traumatic lubricant or control buffer applications to rectum and anus. We measured susceptibility to SHIV_SF162P3_ infection by comparing virus doses needed for rectal infection in twenty-one macaques treated with lubricant or control buffer 30 minutes prior to virus exposure.

**Results:**

Lubricant increased pro-inflammatory cytokines and tissue sloughing while control buffer (phosphate buffered saline; PBS) did not. However, the estimated AID_50_ (50% animal infectious dose) was not different in lubricant- and control buffer-treated macaques (p = 0.4467; logistic regression models).

**Conclusions:**

Although the test lubricant caused acute cytotoxicity in rectal tissues, it did not increase susceptibility to infection in this macaque model. Thus neither the lubricant-induced type/extent of inflammation nor the presence of PQ15 affected infection risk. This study constitutes a first step in the *in vivo* evaluation of lubricants with regards to HIV transmission.

## Introduction

Lubricant use is common in men and in women who practice anal intercourse [1–7]. Personal lubricants are commercially available products marketed to enhance sexual pleasure and to reduce mechanical friction and tissue injury during sex. There is a wide variety of products on the market [8] in diverse gel or liquid formulations, and also co-packaged with condoms. Their chemical composition varies, and can be aqueous-, oil-, or silicone-based. Lubricant ingredients are typically from the “GRAS” list (generally recognized as safe), and do not contain pharmacologically active ingredients.

Recent scientific studies raise concerns about the safety of some personal lubricants [9–13] and their potentially damaging impact on genital epithelial tissues. The rectum may be particularly vulnerable to such damage because it is lined by a single cell layer of columnar epithelial cells, while vaginal or penile tissues have several layers of stratified epithelium. This is relevant for sexually transmitted infections (STI) acquisition, including HIV, since epithelial tissues are barriers for sexually transmitted pathogens.

An emerging hypothesis is that the hyper-osmolality of many water-based lubricants correlates with epithelial tissue damage they induce [9–13]. When epithelial cells are subjected to a hyper-osmolar environment, they release water, leading to cell shrinkage and damage. This can cause breaks in epithelial integrity, and could thereby facilitate entry of invading pathogens, including HIV, to underlying tissues where susceptible HIV target cells reside. Hyper-osmolality is caused by high concentrations of salts in lubricants, even if the chemicals are considered safe in lower concentrations. Results from several studies support an association of lubricant osmolality with cytotoxicity: Aqueous hyper-osmolar lubricants have cytotoxic effects on epithelial cells in *in vitro* models [10,14], in *ex vivo* tissue explants models [9], and in slug mucosal irritation assays [13]. Importantly, the one observational study in humans of a hyper-osmolar mixture of lubricants demonstrated epithelial damage in the human colon [12]. *In vivo* studies of rectal hyper-osmolar lubricant use in mice also documented rectal tissue sloughing, a sign of epithelial tissue damage [11]. In addition to hyper-osmolality, lubricants may have other properties that may affect the genital environment and susceptibility to infection [15]. Polymers like PQ15 added to stabilize aqueous solutions and gels may enhance viral replication, and could thereby enhance sexual virus transmission, including HIV [10]. Thus, there are several potential mechanisms of lubricant-induced tissue damage, but cytotoxicity due to hyper-osmolality has emerged as a leading concern. Despite these data, to date, only one observational study has examined the impact of lubricant use on STI prevalence, and found that rectal lubricant use was associated with increased prevalence of STIs including rectal gonorrhea, chlamydia, and syphilis [16]. Nonetheless, the WHO issued an interim advisory note in 2012 to procurement agencies to avoid purchasing lubricants with osmolality >1,200 mOsm/kg, with pH < 5.5, and containing polymer, PQ15 [17].

Macaques are uniquely suited for studies on HIV risk and prevention because they can be mucosally infected with the HIV homologues, SIV and SHIV. We report the development of a cynomolgus macaque model for evaluation of rectal lubricants, focusing on defining induction, extent, and duration of cytotoxic effects. In this model, HIV acquisition risk was assessed thirty minutes after rectal lubricant use, when cytokine expression was greatest. We selected a water-based lubricant with high hyperosmolality (8,064 mOsm/kg), pH of 4.4, and containing PQ15 [10]. The product was selected before the WHO advisory note was issued; our test lubricant had several properties cautioned against by the WHO, and was well above the natural osmolality of approximately 290 mOsm/kg of human body fluids, as well as that of most previously studied lubricants [10].

## Methods

### Test product

The study lubricant was bought in two batches from a local supermarket, and stored at room temperature as the label recommended. The product is not named in this publication to avoid targeting a product of a single manufacturer. It had the following chemical properties: osmolality: 8,064 mOsm/kg, pH: 4.44 as reported by Begay et al. [10]; ingredients named on package without quantitation: glycerin, propylene glycol, polyquaternium 15, methylparaben, and propylparaben.

### Ethics statement

Macaque experiments were performed according to NIH guidelines [18] and approved by the Institutional Animal Care and Use Committee (IACUC) of the Centers for Disease Control and Prevention (CDC; protocol 2249 KERMONC). Twenty one adult female cynomolgus macaques were purchased from a US vendor (SNBL USA, Ltd; Everett, WA), and housed at CDC. Infection data of an additional eleven cynomolgus macaques using 50 or 250 TCID_50_ SHIV162_p3_ from various unpublished studies were included as historical controls. All animal procedures were performed under anesthesia (10 mg/kg ketamine or 4mg/kg telazol; intramuscular). For housing, macaques were maintained in cages that met (or exceeded) the minimum size requirements as stipulated in the Guide for the Care and Use of Laboratory Animals, 8th Ed. [cage dimensions (inches): 30 (height) x 30 (width) x 30 (length)]. Steps were taken to reduce animal suffering, which included providing enrichment opportunities [e.g. cage features like swings/perches and objects for the macaques to manipulate], an assortment of food selections like fruits, vegetables or seeds, suitable feeding methods (foraging and task-oriented), and humane interactions with caregivers and research staff. Prior to the initiation of virus inoculations, compatible macaques were pair-housed. Once these inoculations were initiated, the macaques were separated into single housing (while permitting eye-contact) with a cage divider to prevent the possibility of SHIV transmission between the macaques. If one macaque remained uninfected during the course of the study, the animal remained separated from other infected macaques, and was not be pair-housed with an infected macaque during the follow-up period. Euthanasia of SHIV infected macaques were accomplished in a humane manner (intravenous pentobarbital) by acceptable techniques as recommended by the American Veterinary Medical Association Guidelines on Euthanasia, 2013, and in accordance with CDC-Atlanta IACUC Policy on Euthanasia. The attending veterinarian and/or trained Animal Resources Branch (ARB) staff verified successful euthanasia by the lack of a heart beat and respiration.

### Time course and sample collection

**Fig 1:**
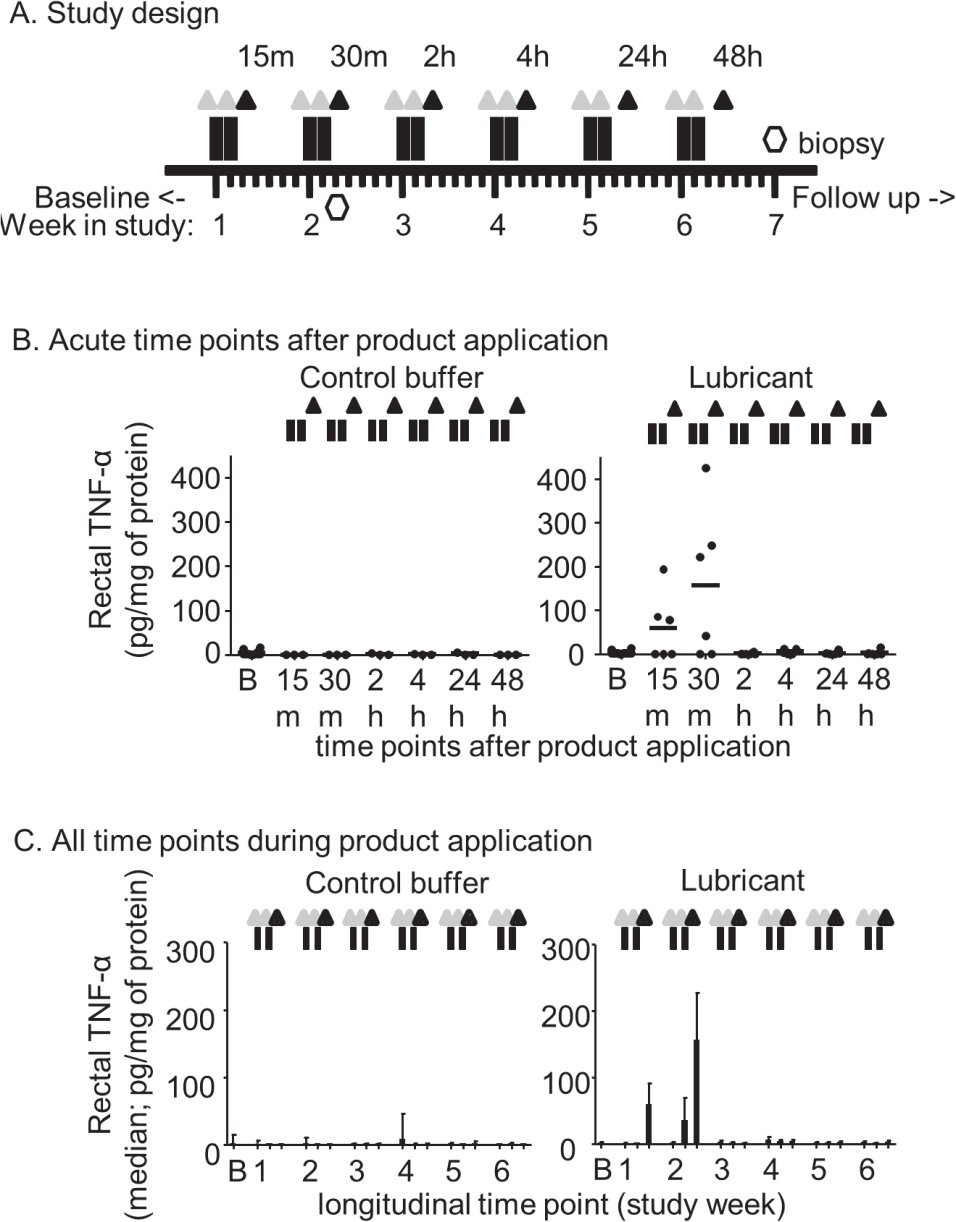
Cytotoxicity study design (Phase I) and induction of pro-inflammatory cytokines. Study design showing the cytotoxicity phase of the study (A); black rectangles = lubricant application; grey triangles = sample collections immediately prior to each product application (longitudinal time points); black triangles = samples taken 15 minutes to 48 hours after product application (acute time points); open hexagons = rectal biopsies, taken from one animal at 30 minutes post lubricant-application, and from one animal a week after last lubricant application; m = minutes; h = hours; B. Induction of pro-inflammatory cytokine TNF-α at acute time points (15 or 30 m, and 2, 4, 24, or 48 h post-product application); circles represent individual macaques; C. Induction of pro-inflammatory cytokine TNF-α at all time points during 6 weeks of product application; medians and ranges are graphed.

For cytotoxicity studies, macaques received non-traumatic applications of lubricant (n = 6) or phosphate buffered saline (PBS; n = 3) twice a week in a study design shown in Fig. 1, A. PBS was chosen because the test lubricant was a liquid, not a gel. Using a 10cc syringe attached to a flexible sterile 8-gauge feeding tube, 3mls of lubricant or PBS were gently infused into the rectum 2–4cm beyond the anal sphincter. This was followed by gentle application of another 2mls to the anal area with a swab. Samples were acquired at baseline for three weeks, and then as shown in Fig. 1, A. Rectal pH was determined by rolling a polyester swab against the rectal wall, approximately 3cm beyond the anal sphincter, and dabbing it onto a pH indicator strip. For cytokine measurements, Merocel sponges (Medtronic, Jacksonville, FL) pre-wet with sterile PBS were placed for 2 minutes in the rectum approximately 3cm beyond the anal sphincter, and allowed to absorb rectal secretions. Post-collection, samples were kept on ice and processed immediately using a mild extraction buffer with the following protease inhibitors: trypsin-chymotrypsin inhibitor (5μg/mL), E-64 (14μM) and bestatin (130μM) (Sigma, St. Louis, MO). Rectal lavage was obtained by injecting 8ml of PBS into the rectum of the macaques positioned at a 30° angle to reduce trauma during collection [19]. PBS was gently recovered after 20–30 seconds.

### Epithelial tissue sloughing assay, associated blood

Rectal lavages were immediately assessed for epithelial sloughing (epithelial sheets ≥3mm in any dimension) [18], and associated blood using a dissecting microscope. To reduce fecal background, lavages were PBS-diluted and transferred to clean plates. High resolution images were obtained using an Axiophot upright microscope (Carl Zeiss, Oberkochen, Germany). Samples were also examined by hematoxylin and eosin (H&E) staining.

### Microflora

Rectal swabs were collected, placed in Port-a-Cul tubes (BD, Franklin Lakes, NJ), and the microflora analyzed (for types and total numbers of cultivable bacteria) at Magee-Womens Research Institute (Pittsburgh, PA).

### Histopathological Evaluation and Immunohistochemical (IHC) Assays

Pinch biopsies from 3–5cm beyond the anal sphincter were fixed in formaldehyde, and stored at room temperature in 70% ethanol. H&E stains of the rectal biopsies were evaluated for histopathological changes as previously described [19]. IHC assays were performed on 3μm tissue sections using antibodies against CD68, CD3 (DAKO, Carinteria, CA), or CD79 (Santa Cruz Biotechnology, Inc). Negative controls consisted of sequential tissue sections incubated with either normal rabbit (CD3) or mouse serum (CD79, CD68).

### Cytokine measurements

Rectal secretions eluted from Merocel sponges were assessed for cytokines, chemokines, and other inflammatory markers (IL-1β, IL-1Ra, IL-2, IL-4, IL-5, IL-6, IL-8, IL-10, IL-12, IL-13, IL-15, IL-17, IL-18, G-CSF, GM-CSF, IFN-γ, TNF-a, MIP1α, MIP1β, MCP1, TGFA, VEGF, sCD40) using Luminex bead technology (Bioplex, Biorad, Hercules, CA) and a non-human primate cytokine bead panel (Millipore, Billerica, MA). The measured proteins were normalized against total soluble protein levels in the rectal secretions determined using Quick Start Bradford protein assay (Biorad, Hercules, CA).

### Virus challenges and virus detection

To determine susceptibility to infection, we compared virus doses needed for infection in 21 lubricant- or control buffer-treated cynomolgus macaques using an animal model modified from earlier reports [20,21]. Thirty minutes after lubricant application, animals were exposed rectally to virus at varying doses (1.25–25,000 tissue culture infectious doses [TCID_50_]) of SHIV_SF162P3_ obtained from NIH [22], propagated in cynomolgus macaque PBMCs. First, we challenged the PBS-treated animals in sets of ten and nine macaques each. The uninfected macaques (n = 12; rested for at least 6 weeks) and two additional macaques were then lubricant-treated and exposed to various virus doses. Plasma viral RNA was measured by reverse-transcriptase polymerase chain reaction (PCR) with a 50 copies/ml detection limit [23]. In addition to viral RNA detection on two consecutive blood draws, in-house SHIV proviral real-time PCR assays and EIA detection of HIV envelope-specific antibodies (Bio-Rad HIV-1/HIV-2 Plus O EIA, Hercules, CA) confirmed infections. For viral RNA determination in the rectal lavages, samples were quick spun at low speeds to remove fecal debris, and supernatants stored at −80°C until viral RNA determination.

### Statistical evaluation

For cytokine analyses, we converted all cytokine measurements below the limit of detection (LOD) to the actual LOD value given for that particular cytokine in the manufacturer’s kit manual. For each study arm, we compared the geometric mean (GM) of baseline measurements to those at acute time points (15m, 30m, 2h, 4h) post-PBS or lubricant application. To ascertain differences between the two arms, we calculated the ratio of the GM ratios in the two arms normalized to baseline. Statistical differences were estimated using 2-tailed unpaired t-tests. To compare sloughing and rectal pH, with and without lubricant application, a 2-sided Fisher’s exact probability test and a 2-tailed Mann-Whitney test were used, respectively. Statistical comparisons could not be made for bleeding because of the limited data set. We calculated animal infectious doses (AID_50_) by modeling HIV infection as a function of the log10 TCID_50_. The log10 AID_50_ values were estimated from the logistic regression model for the lubricant and control groups, and compared using an unpaired t-test.

## Results

### Cytotoxicity evaluation

Fig. 1, A shows the study design and sample collection schedule for assessing longitudinal or acute cytotoxic lubricant effects over six treatment weeks with twice weekly product applications on consecutive days in nine macaques (n = 6 lubricant-treated, n = 3 PBS-treated controls). “Longitudinal samples” refer to those collected immediately prior to each product application in order to analyze long lasting or cumulative effects, while “acute samples” refer to those collected to assess acute effects 15 minutes to 48 hours after the second weekly product application. To determine the effects of lubricant use on inflammation, we analyzed cytokines and other inflammatory markers from rectal secretions. Numerous cytokines peaked significantly at acute time points post-lubrication [TNF-α (p = 0.024), IL-12 (p = 0.016), IL-15 (p = 0.015), G-CSF (p = 0.013), sCD40 (p = 0.050), IL-4 (p = 0.009), MCP-1 (p = 0.026), MIP-1α (p = 0.014), and IL-1β (p = 0.008); Table 1]. Fig. 1, B and C show TNF-α induction as an example for a pro-inflammatory cytokine upregulated at acute time points after lubricant use, but not longitudinally during the six weeks of treatment. Rectal TNF-α peaked 30 minutes after the second weekly lubricant application. Cytokines were not upregulated compared to baseline in longitudinal samples collected over six weeks, suggesting no persistent or cumulative effect of frequent lubricant use on these inflammatory markers.

**Table 1:** Cytokine concentration in rectal lavages at acute time points post lubricant/control buffer application.

Cytokine	Conc^1^ (95% CI) for controls at acute time points; pg/(mg of total proteins)	Conc^1^ (95% CI) for lubricant-treated animals at acute time points; pg/(mg of total proteins)	p-value (control arm versus lubricant-treated arm)
**IL-1β**	0.3638 (0.10,1.23)	3.2008 (1.76,5.79)	**0.008**
**IL-4**	1.016 (0.34,3.02)	5.813 (3.11,10.84)	**0.009**
**G-CSF**	0.0680 (0.004,1.11)	1.624 (0.43,6.09)	**0.013**
**MIP-1α**	1.443 (0.48,4.26)	3.688 (1.76,7.70)	**0.014**
**IL-15**	1.5786 (0.82,3.03)	3.1443 (2.04,4.85)	**0.015**
**IL-12**	0.8424 (0.28,2.49)	3.2886 (1.60,6.72)	**0.016**
**TNF-α**	0.091 (0.006,1.30)	3.245 (0.86,12.23)	**0.024**
**MCP-1**	1.948 (0.72,5.25)	11.364 (5.97,21.6)	**0.026**
**sCD40**	0.669 (0.13,3.75)	2.534 (0.80,8.00)	**0.050**
**IFN-γ**	0.090 (0.004,1.66)	0.611 (0.12,2.96)	0.093
**TGFα**	8.592 (4.62,15.97)	13.303 (8.62,20.53)	0.137
**IL-8**	11.303 (4.20,30.40)	63.247 (31.83,125.6)	0.179
**IL-1Ra**	27.769 (16.94,45.50)	86.328 (60.88,122.4)	0.199
**VEGF**	18.722 (7.51,46.64)	32.394 (17.48,60.01)	0.421
**IL-18**	26.935 (17.42,41.65)	25.907 (19.05,35.23)	0.732

The p-values were calculated using unpaired t-tests (see Materials & Methods for details); the statistically significant ones are indicated in bold; CI = confidence interval.

^1^We calculated the geometric means (GMs) of cytokines concentrations as shown, combining the measurements at 15m, 30m, 2- and 4-h acute time points. Levels of eight other cytokines were determined (see Methods) but many of the data points were below the assay limit of detection, and not suitable for accurate statistical analyses

### Epithelial sloughing and blood

**Fig 2:**
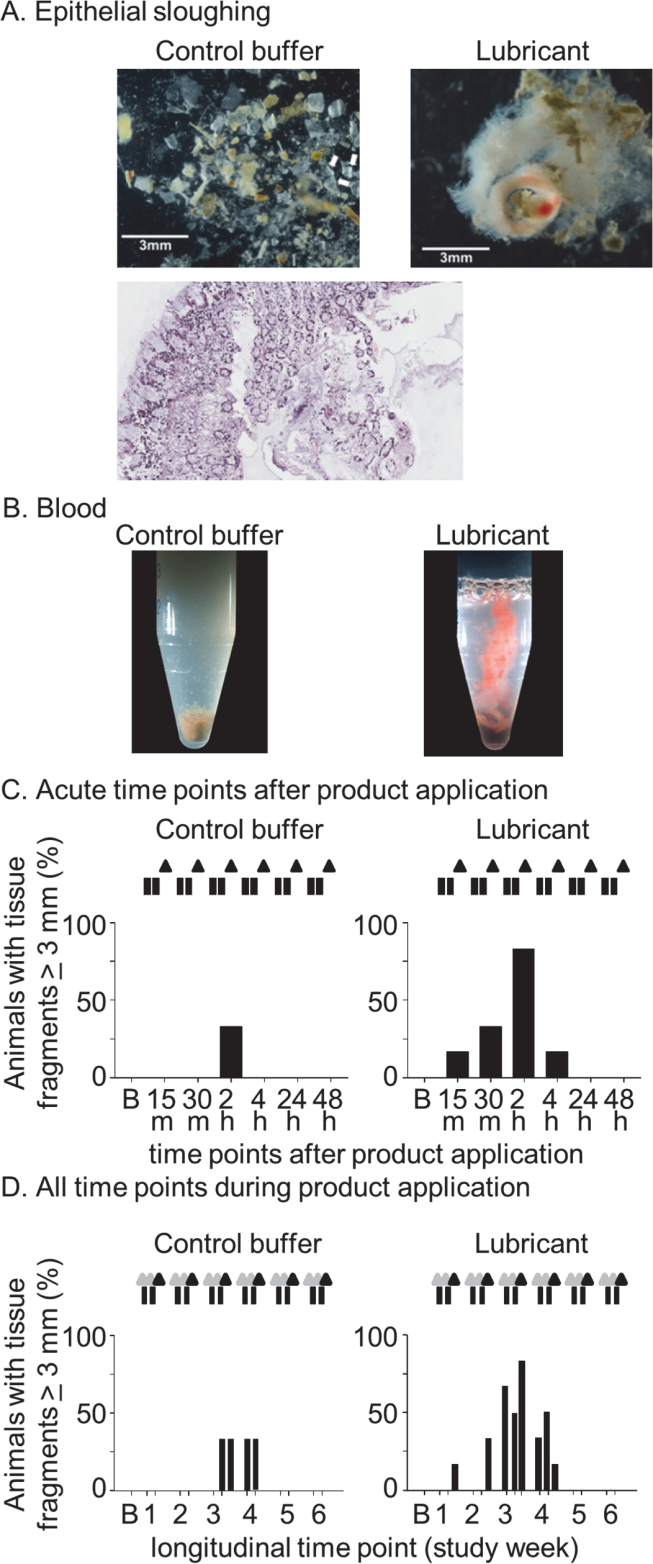
Epithelial sloughing and blood. A. Lubricant induces rectal shedding of epithelial cells. Examples of epithelial sloughing in a control- (top left) and lubricant-treated animal (top right). The lower panel shows a representative H&E stain of sloughed rectal epithelial cells (20x); B. Blood associated with rectal washes; photographs of microfuges containing rectal lavages; C,D. Epithelial sloughing measured at acute time points collected after the 2nd weekly lubricant application (C), and those measured over the entire study (D). The panel D in this figure shows three collections per week (day 1, pre-lubricant; day 2 pre-lubricant; day 2 post-lubricant), as indicated in Fig. 1.

Tissue damage was examined by determining epithelial sloughing and associated blood in rectal lavages. Fig. 2, A shows photographs of epithelial sheets with size >3mm in any dimension, harvested from the rectum of a lubricant-treated macaque. The size of the sloughed cellular material varied from 3mm to 20mm. Controls exhibited a lesser degree of epithelial shedding (<3mm in any dimension), which is considered normal [18]. Histological examination showed that the sloughed material was of cellular origin, consistent with the shedding of epithelium (Fig. 2, A; lower panel). Blood associated with sloughed tissues was documented by visual and microscopic examination of lavages (Fig. 2, B). Blood was only associated with epithelial sheets following lubricant application, and not in control animals. Fig. 2, C and D display the frequency of detected tissue fragments > 3mm in all evaluated macaques. Sloughing occurred in five of six macaques 2 hours post-lubricant treatment, while only one of the three control macaques had signs of this type of tissue sloughing at any acute time point. The difference in sloughing at acute time points (Fig. 2, C) in the two study arms was not statistically significant (p = 0.1379; 2-tailed Fisher’s exact probability test). Fig. 2, D that displays all times points of collection (longitudinal) shows that sloughing did not increase over time. However maximum shedding of epithelium was seen in week 3 where all three collection points (over two days) showed sloughing in up to 5 out of 6 lubricant-treated animals. At this point, sloughing was seen on day 1 (pre-lubricant) and day 2 (post-lubricant) in only 1 out of 3 controls. The sloughing was also evident in week 4 but in fewer macaques. S1 Fig. shows the time course of visually observed blood associated with shed tissue fragments. Bleeding was first noted in some animals after week 1. Following repeated lubricant applications (weeks 4–6), blood in some lavage samples became discernible by eye. It peaked at 4h post-application and was detected in 67% of lubricant animals, and in none of the controls.

### Histology

**Fig 3:**
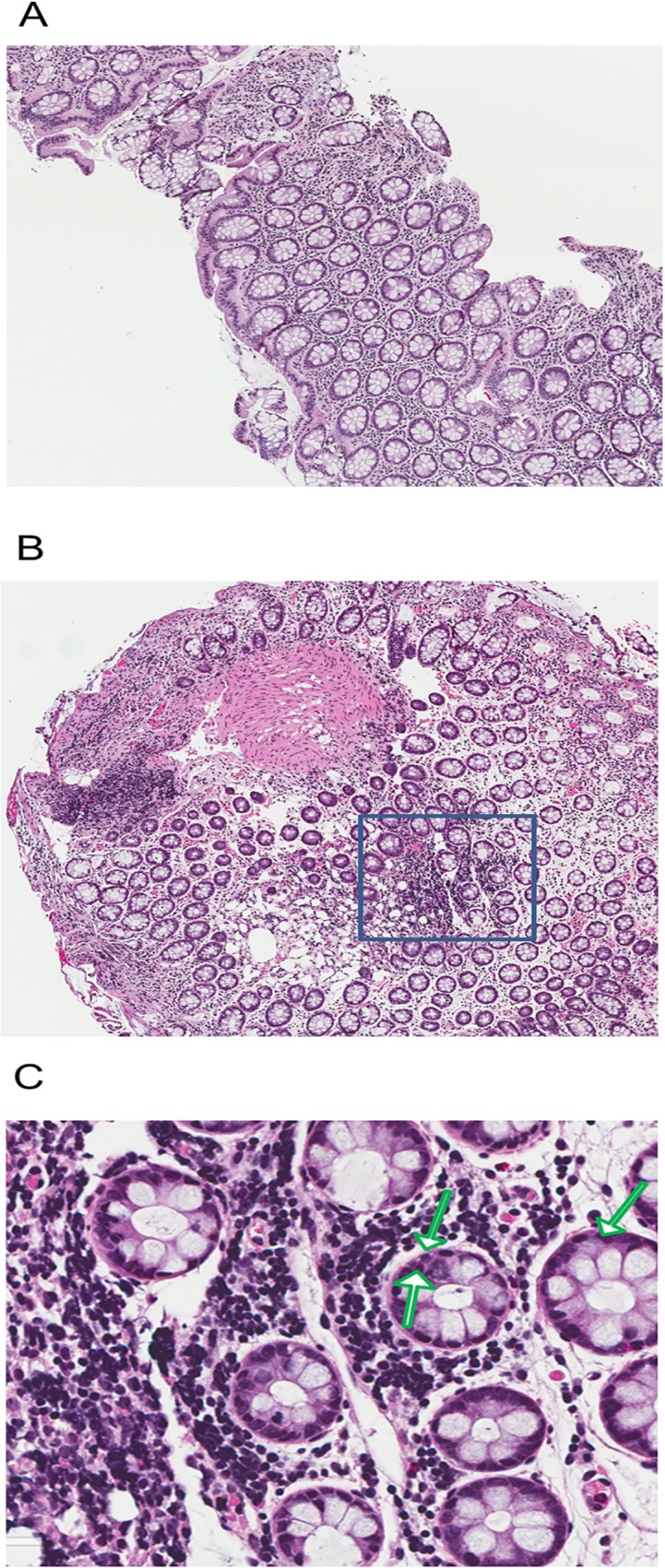
Hematoxylin and eosin stain (20x) of rectal biopsies. Showing biopsies from one animal (ID: 604962) collected before lubricant application (A) and 30 minutes after (B) product application; B shows focal infiltrates of inflammatory cells (square box), predominately mononuclear, seen in the lamina propria; there is no disruption of architecture. C is a magnified section (30x) of the square box with the green arrows showing mononuclear cells.

Rectal biopsies were taken from one animal 30 min after the final lubricant application, and they showed focal infiltrates of inflammatory cells (Fig. 3), predominantly mononuclear, seen in the lamina propria; there was no disruption of architecture. Biopsies collected in one animal a week after final lubricant application did not demonstrate significant tissue damage or inflammatory cell infiltration (H&E and IHC staining with CD3, CD6 or CD79) in lubricant-treated macaques (data not shown)

### Rectal microflora and pH

Lubricant application did not lead to a significant reduction in average levels of bacteria harvested from rectal swabs, analyzed in a subset of 5 animals (S2 Fig.) showing that the lubricant did not directly reduce rectal microflora. The type and composition of the microflora (n = 5) varied largely over time in both lubricant-treated and untreated macaques (data not shown). Due to a limited data set, statistical analyses were not performed on differences in microflora type/composition. The median rectal pH was 7.9 (S3 Fig.), both before and after lubricant treatment (p = 0.4230; unpaired 2-tailed Mann-Whitney test), despite the study lubricant’s pH of 4.4.

### SHIV challenges and infections

**Fig 4:**
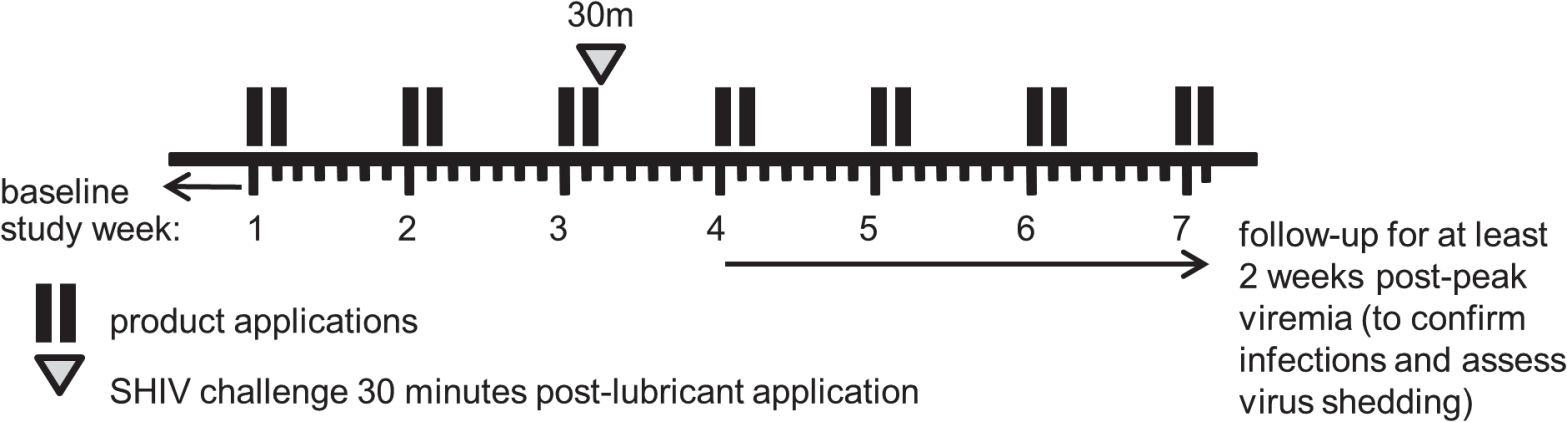
Challenge study design (Phase II). Lubricant was applied non-traumatically for three weeks, on two consecutive days per week. The animal was challenged with SHIV_SF162p3_ 30 minutes after the sixth lubricant application (open triangle). To check for plasma viremia and virus shedding, samples were collected for at least 2 weeks post-peak viremia. Not shown are baseline blood draws that were performed before the first lubricant application to ensure that the animal is not infected.

In order to determine whether the observed, lubricant-induced cytotoxicity may affect HIV risk, we used the cynomolgus macaque model to study rectal SHIV_SF162P3_ infection risk. Fig. 4 shows the study design; the lubricant was applied non-traumatically over three weeks, on two consecutive days per week, and virus challenges occurred 30 minutes after the sixth lubricant application since the cytokine levels peaked at 30 min post-application. We determined relative susceptibility to infection by comparing the SHIV_SF162P3_ doses (AID_50_) needed for infection in lubricant- or control buffer-treated macaques in two study arms. Macaques were first exposed to low doses of virus, and if they remained uninfected, they were rested, and then re-enrolled at higher doses until infection occurred. The virus exposure schedule for individual macaques can be seen in S1 Table. In total, 21 macaques were challenged with various doses until 15 infections occurred in a total of 51 exposures (controls = 7, lubricant = 8). Table 2 summarizes the number of virus exposures and outcomes. Unexpectedly, only two out of nine lubricant-treated animals were infected at doses >2,500 TCID_50._ In contrast, six of the seven macaques were infected in the control arm at those higher doses. In this study, we also used data from historical controls, i.e., from 11 macaques exposed to SHIV_SF162P3_ at 50 or 250 TCID_50_s, with one infection occurring at each dose.

**Table 2:** SHIV162p3 challenge doses and infection.

Virus dose (TCID_50_)	PBS-treated^1^	Lube-treated
**25000**	+	00
**5,000**	++	00+
**2,500**	0+++	000+
**1,250**	0000	000++
**500**	00	0000++
**250**	00000000++	000++
**50**	0000+	00
**12.5**	00	00
**1.25**		000

‘+’ = animal infected at the indicated dose;

‘0’ = animal not infected;

PBS = phosphate buffered saline; TCID_50_ = tissue culture 50% infectious dose. The challenges were performed in six sets of macaques; sets1 and 2 were phosphate buffered saline (PBS)-treated controls; sets 3, 4, 5 and 6 were lubricant-treated.

^1^Historical data from 5 uninfected and 1 infected animals were included at 250 TCID_50_, and 4 uninfected and 1 infected animals at 50 TCID_50_; these animals were non-PBS-treated

### Risk of SHIV transmission

In order to define SHIV susceptibility to infection we used logistic regression models to estimate the AID_50_. Contrary to the hypothesis that the lubricant may increase susceptibility to infection, lubricant-treated macaques did not uniformly become infected at lower virus doses. The estimated AID_50_ was 1.96x10^5^ in the lubricant-treated arm and 1.7x10^3^ in the controls (S4 Fig.). The 95% confidence interval (CI) for AID_50_ was 1.0–4.5x10^10^ in lubricant-treated macaques and 414–7165 in controls. The AID_50_ in the lubricant arm was not as well-defined as the control arm because of a lack of as many infections at SHIV doses >2,500 TCID_50_. The estimated AID_50_ ratio of lubricant-treated macaques to controls was 114 (95% CI: 4.0x10^−3^–3.5 x 10^6^), which was not statistically significantly different (p = 0.4467).

### Plasma viremia and rectal virus shedding

**Fig 5:**
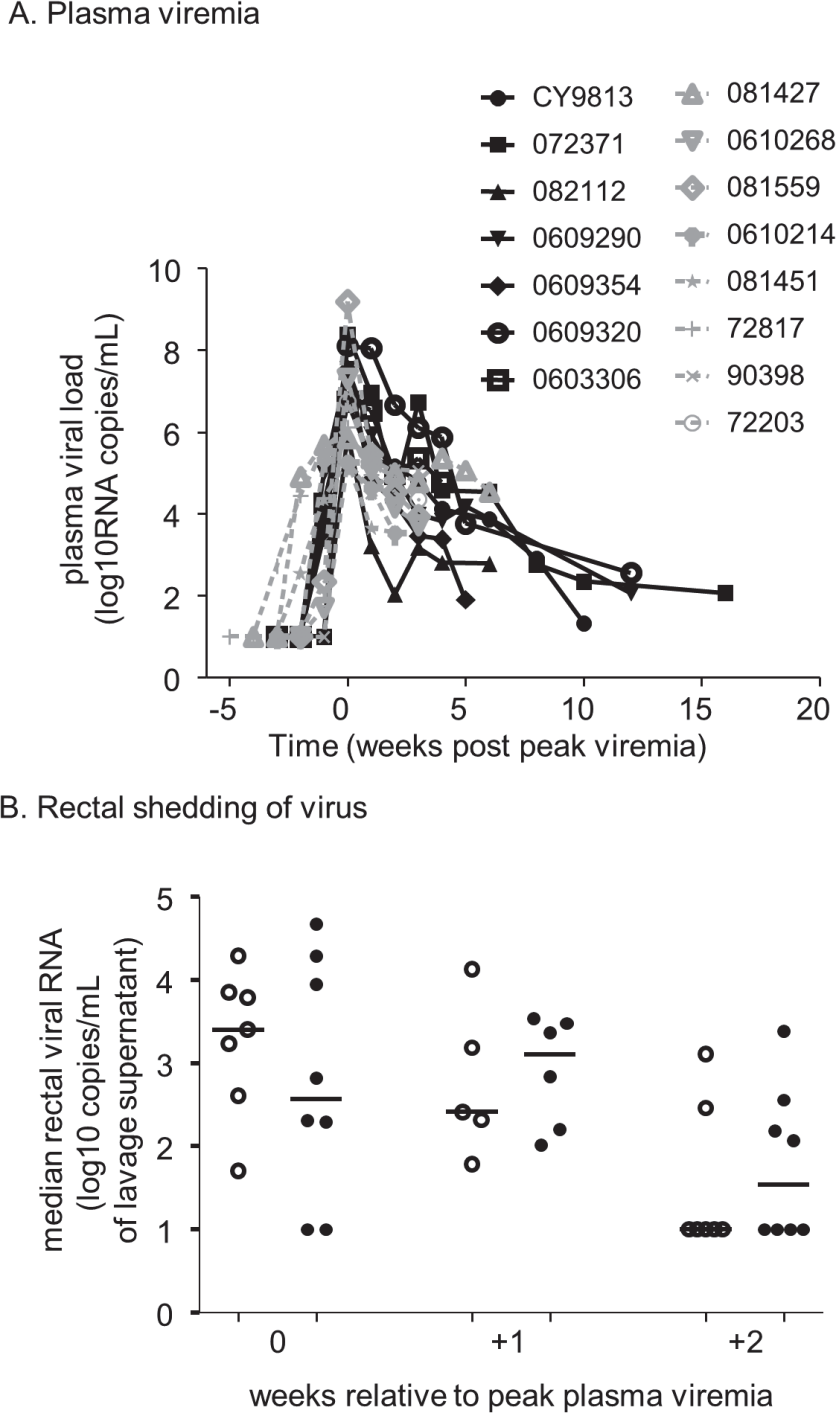
Plasma and rectal SHIV RNA. A. Plasma SHIV RNA levels measured in control buffer-treated (black symbols; solid lines) and lubricant-treated (grey symbols; broken lines) animals. B. SHIV RNA levels measured in rectal secretions, in control buffer-treated (open symbols) and lubricant-treated (closed symbols) animals; 0 = time of peak plasma viremia.

After infection, and as shown in the study design (Fig. 4), we continued to apply lubricant for at least 2 weeks to determine any effects on the course of SHIV infection and on genital shedding of virus. Fig. 5 displays the course of SHIV_SF162P3_ infection over time. The median peak plasma viremia was 3.5 x10^7^ viral copies/mL in the control arm and 1x10^6^ viral copies/mL in the lubricant arm, and was not significantly different (p = 0.1206; unpaired, two-tailed Mann-Whitney test). Additionally, viral RNA levels in the two arms were compared at a subsequent time point post-virus challenge when data points from all animals were available (5 weeks). Again, we found the viral RNA levels did not differ significantly (p = 0.9551; unpaired, two-tailed Mann-Whitney test). Virus shedding in rectal secretions is shown in Fig. 5, B at the time of peak plasma viremia and during the ensuing two weeks during continued rectal lubricant application. No statistically significant differences in rectal virus shedding were observed (two-tailed Mann-Whitney test) at these time points.

## Discussion

The evaluation of one highly osmolar lubricant revealed acute rectal cytotoxicity in cynomolgus macaques, as also observed by others in humans, mice, and *in vitro* systems [9–13]. When we tested the hypothesis using a model of varying virus doses that lubricant-induced cytotoxicity would increase rectal SHIV infection risk, we did not see evidence of heightened susceptibility to infection. The most likely explanation is that extent, type, and duration of cytotoxicity and inflammation were not sufficient for detectable risk increases in this model. In particular, inflammation and cytokine induction were largely short-lived (peaking at 30 minutes, and subsiding within two hours after lubricant use), and less pronounced than we have previously observed during rectal co-infection with *Chlamydia trachomatis* in a similar macaque model [24]. Other cytotoxicity parameters such as bleeding and tissue sloughing were documented after lubricant use, but were not significantly upregulated compared to buffer-treated macaques.

Despite the low pH of the test product, there were no significant changes in the pH of the anal-rectal compartment perhaps reflecting the weak buffering capacity of the lubricant. The limited data set on microflora showed no effects of lubricant application on microbial numbers. Rectal biopsies taken in one animal 30 minutes post-product application showed mild inflammation, whereas those taken 1 week after application did not show any inflammation. These findings may be best explained by the ability of the rectum to rapidly repair itself after lubricant-induced damage is inflicted, and cytotoxicity may not rise to a level that measurably affects SHIV transmission. Recently, Dezzutti et al. tested a lubricant from the same product family using *ex vivo* explants for its ability to enhance HIV-1 uptake and did not find evidence for enhanced infection [9]. Future studies with rectal biopsies could involve more in-depth analyses of immune infiltrates including study of cellular activation or death post-lubricant application.

The overall lack of risk enhancement is perhaps not surprising in the context of nonoxynol-9 (N9), a surfactant that has been studied for HIV risk impact [9]. N9 causes acute cytotoxicity when rectally applied to macaques [25] but shows no long-lasting effects, similar to our study findings. In humans, the impact of N9 might be dependent on frequency of use (shown in only one study by Van Damme et al. [26]) but such correlations could not be determined in our study because we did not model high frequency use.

It is possible that a different study design for lubricant application (e.g., timing and duration of lubricant application and sampling, frequency, and trauma during use of the product) and for risk assessment may have yielded different results. Repeated lubricant applications were modeled after hypothesized human use on two weekend days. Limiting lubricant applications to two days/week was also necessary to limit repeated anesthesia applications per week over several weeks due to the necessary fasting, and associated weight loss in macaques. This allowed us to give two lubricant applications, and use the third anesthesia for sample collections. A higher lubricant application frequency might have increased the measurable damage, although there was no evidence of lasting damage on the second day of the weekly applications. It is possible that cytotoxicity peaked later than 30 minutes after lubricant applications but before our next time points. We chose the 30 minute time point for SHIV application because cytokine induction was maximal at the time point, and also because a 30 minute interval between lubricant application and intercourse with virus exposure was deemed a relevant scenario for human lubricant use. Also, we used non-traumatic product application (without use of semen) to achieve humane animal experimentation, but it does not entirely represent human lubricant use during sex. It is possible that lubricant effects combined with mechanical trauma or semen during sex would affect cytotoxicity and HIV risk more so than detected here. Taken together, our study design and results may underestimate the effect of lubricant on cytotoxicity and also SHIV risk. However, it is also possible that we underestimated protective effects of lubricants in this model. Personal lubricants can protect from mechanical tissue damage during sex. Sex was not modeled in this animal model, and protective properties could not be assessed. They may outweigh the cytotoxic effects observed in this study.

One unexpected finding was a seemingly wider range of susceptibilities to infection in lubricant- compared to buffer-treated macaques. We used a model of varying virus doses similar to Chenine et al [20] who reported a 17-fold difference in virus doses needed to infect *Schistosoma mansoni*-infected macaques compared to *S*. *mansoni*-negative animals. Hence, the model can provide great sensitivity in detecting enhanced risk. We started by establishing a virus dose infection curve in control macaques, and exposed uninfected macaques to higher doses. Unexpectedly, several macaques remained uninfected at the highest doses (undiluted stocks), and those resistant macaques were subsequently placed in the lubricant-treated group, which is a limitation of the study. To address this, we added naive macaques to the lubricant group, and they also remained uninfected at undiluted stock doses (e.g., animal ID = 70782; S1 Table). To increase statistical power, non-PBS-treated cynomolgus macaques were included as historical controls. Other limitations are that we cannot reproducibly test in non-human primates the impact of lubricant on cell-to-cell transmission, and that the cynomolgus macaque model of HIV susceptibility and transmission is not as well defined as rhesus or pigtail macaque models. Also, we cannot confirm whether cytotoxicity was evident in macaques at the time of virus challenges.

Several recent scientific studies have supported the hypothesis that hyper-osmolality of personal lubricants may be the leading cause of cytotoxicity [9,10,12]. Since we evaluated only a single product, we cannot conclude whether hyper-osmolality or other physical and chemical properties caused cytotoxicity. Subsequent to our selection of study lubricant the WHO issued an advisory note to avoid procurement of lubricant products with osmolality >1,200 mOsm/kg, with pH < 5.5, and containing PQ15 [17], which were properties that our study lubricant possessed [9,10]. More systematic evaluations, perhaps in species other than macaques with better high-throughput capacity, are needed to determine the chemical basis for cytotoxicity.

In summary, this study constitutes a first step in the *in vivo* evaluation of lubricants with regards to HIV transmission. A highly osmolar lubricant did not increase SHIV transmission risk in cynomolgus macaques. This was reassuring considering the widespread use of products with these properties. However our study had limitations, and we do not consider this study as a sufficient scientific basis to draw definitive conclusions or to make recommendations on lubricant use and HIV prevention. In the interim, correct and consistent use of condoms is recommended to prevent exposure to pathogens, including HIV, which the CDC and WHO recommend during sexual intercourse [27][28].

## Supporting Information

S1 ARRIVE Checklist (PDF)

S1 Fig Blood associated with rectal washes. The percentage of macaques with visible blood in their rectal washes is plotted for time points acutely after product application (A), or for all time points of specimen collection (B).
(TIF)

S2 Fig Levels of harvested bacteria from rectal swabs. We found no significant difference (unpaired, two-tailed t-test) between the two arms; bacteria were cultured and counted from rectal swabs. “B” refers to baseline; three independent time points were analyzed. “+” refers to product-treated, six time points were analyzed. Data was combined from two controls, and three lubricant-treated animals. Lines are medians; open circles are from controls, filled circles from the lubricant group.
(TIF)

S3 Fig Analysis of rectal pH. Application of a lubricant with pH of 4.4 does not significantly alter rectal pH. Rectal fluids were collected with a cotton swab, and immediately applied to pH strips. “B” baseline (Six independent time points); only “acute” time points (after second weekly product application) are shown; “m” minutes, “h” hours. Mean and SEM are shown; the schematic refers to study design shown in Fig. 1.
(TIF)

S4 Fig Probability of SHIV infection using a logistic regression models (including data from historical controls). The estimated AID50 ratio (114) between the study arms did not show statistically significant difference (p = 0.45); shown here are the dose curves for the control buffer-treated (black line) and lubricant-exposed (grey line) animals; the log10 value for the lubricant arm was extrapolated.
(TIF)

S1 Table SHIV challenge design showing the animal IDs. Sets1 and 2 were phosphate buffered saline (PBS)-treated controls (c); sets 3, 4, 5 and 6 were lubricant-treated (L); ‘+’ = animal infected; ‘−’ = animal not infected; historical controls are not shown here. The virus doses (in TCID50) are indicated in parenthesis next to every SHIV challenge. During the cytotoxicity testing phase of the study, animals 610214, 610268, and 607780 were PBS-treated, and animals CY9813, 609290, 609354, 603306, 609320, and 604962 were lubricant-treated.
(PDF)

## References

[1] Jozkowski KN, Herbenick D, Schick V, Reece M, Sanders SA, et al (2013) Women's perceptions about lubricant use and vaginal wetness during sexual activities. J Sex Med 10: 484–492. 10.1111/jsm.12022 23211029

[2] Calabrese SK, Rosenberger JG, Schick VR, Novak DS, Reece M (2013) An event-level comparison of risk-related sexual practices between black and other-race men who have sex with men: condoms, semen, lubricant, and rectal douching. AIDS Patient Care STDS 27: 77–84. 10.1089/apc.2012.0355 23373663 PMC3565550

[3] Carballo-Dieguez A, Stein Z, Saez H, Dolezal C, Nieves-Rosa L, et al (2000) Frequent use of lubricants for anal sex among men who have sex with men: the HIV prevention potential of a microbicidal gel. Am J Public Health 90: 1117–1121. 10897191 10.2105/ajph.90.7.1117 PMC1446289

[4] Kinsler JJ, Galea JT, Peinado J, Segura P, Montano SM, et al (2010) Lubricant use among men who have sex with men reporting receptive anal intercourse in Peru: implications for rectal microbicides as an HIV prevention strategy. Int J STD AIDS 21: 567–572. 10.1258/ijsa.2010.010134 20975090

[5] Gross M, Buchbinder SP, Celum C, Heagerty P, Seage GR 3rd (1998) Rectal microbicides for U.S. gay men. Are clinical trials needed? Are they feasible? HIVNET Vaccine Preparedness Study Protocol Team. Sex Transm Dis 25: 296–302. 9662763 10.1097/00007435-199807000-00005

[6] Javanbakht M, Murphy R, Gorbach P, LeBlanc MA, Pickett J (2010) Preference and practices relating to lubricant use during anal intercourse: implications for rectal microbicides. Sex Health 7: 193–198. 10.1071/SH09062 20465986

[7] Herbenick D, Schick V, Reece M, Sanders SA, Smith N, et al (2013) Characteristics of condom and lubricant use among a nationally representative probability sample of adults ages 18–59 in the United States. J Sex Med 10: 474–483. 10.1111/jsm.12021 23346924

[8] Pickett J (2008) Less Silence, more science: Advocacy to Make Rectal Microbicides a Reality. http://www.rectalmicrobicides.org/docs/IRMA%20FinalB&WWeb.pdf. Accessed 10 February 2015.

[9] Dezzutti CS, Brown ER, Moncla B, Russo J, Cost M, et al (2012) Is Wetter Better? An Evaluation of Over-the-Counter Personal Lubricants for Safety and Anti-HIV-1 Activity. PLoS One 7: e48328 10.1371/journal.pone.0048328 23144863 PMC3492332

[10] Begay O, Jean-Pierre N, Abraham CJ, Chudolij A, Seidor S, et al (2011) Identification of personal lubricants that can cause rectal epithelial cell damage and enhance HIV type 1 replication in vitro. AIDS Res Hum Retroviruses 27: 1019–1024. 10.1089/AID.2010.0252 21309617 PMC3161103

[11] Sudol KM, Phillips DM (2004) Relative safety of sexual lubricants for rectal intercourse. Sex Transm Dis 31: 346–349. 15167643 10.1097/00007435-200406000-00005

[12] Fuchs EJ, Lee LA, Torbenson MS, Parsons TL, Bakshi RP, et al (2007) Hyperosmolar sexual lubricant causes epithelial damage in the distal colon: potential implication for HIV transmission. J Infect Dis 195: 703–710. 17262713 10.1086/511279

[13] Adriaens E, Remon JP (2008) Mucosal irritation potential of personal lubricants relates to product osmolality as detected by the slug mucosal irritation assay. Sex Transm Dis 35: 512–516. 10.1097/OLQ.0b013e3181644669 18356773

[14] Maguire RA, Bergman N, Phillips DM (2001) Comparison of microbicides for efficacy in protecting mice against vaginal challenge with herpes simplex virus type 2, cytotoxicity, antibacterial properties, and sperm immobilization. Sex Transm Dis 28: 259–265. 11354263 10.1097/00007435-200105000-00003

[15] Nguyen D, Lee H, Poast J, Cloyd MW, Baron S (2004) Preventing sexual transmission of HIV: anti-HIV bioregulatory and homeostatic components of commercial sexual lubricants. J Biol Regul Homeost Agents 18: 268–274. 15786693

[16] Gorbach PM, Weiss RE, Fuchs E, Jeffries RA, Hezerah M, et al (2012) The slippery slope: lubricant use and rectal sexually transmitted infections: a newly identified risk. Sex Transm Dis 39: 59–64. 10.1097/OLQ.0b013e318235502b 22183849 PMC3244680

[17] WHO, FHI, UNFPA (2012) Use and procurement of additional lubricants for male and female condoms: WHO/UNFPA/FHI. WHO reference number: WHO/RHR/12.33. http://apps.who.int/iris/bitstream/10665/76580/1/WHO_RHR_12.33_eng.pdf. Accessed 2015 Feb 10.

[18] Patton DL, Sweeney YT, Paul KJ (2009) A summary of preclinical topical microbicide rectal safety and efficacy evaluations in a pigtailed macaque model. Sex Transm Dis 36: 350–356. 10.1097/OLQ.0b013e318195c31a 19556929 PMC2749653

[19] Shieh WJ, Blau DM, Denison AM, Deleon-Carnes M, Adem P, et al (2010) 2009 pandemic influenza A (H1N1): pathology and pathogenesis of 100 fatal cases in the United States. Am J Pathol 177: 166–175. 10.2353/ajpath.2010.100115 20508031 PMC2893660

[20] Chenine AL, Shai-Kobiler E, Steele LN, Ong H, Augostini P, et al (2008) Acute Schistosoma mansoni infection increases susceptibility to systemic SHIV clade C infection in rhesus macaques after mucosal virus exposure. PLoS Negl Trop Dis 2: e265 10.1371/journal.pntd.0000265 18648516 PMC2447882

[21] Spouge JL (1992) Statistical analysis of sparse infection data and its implications for retroviral treatment trials in primates. Proc Natl Acad Sci U S A 89: 7581–7585. 1323844 10.1073/pnas.89.16.7581 PMC49754

[22] Harouse JM, Gettie A, Eshetu T, Tan RC, Bohm R, et al (2001) Mucosal transmission and induction of simian AIDS by CCR5-specific simian/human immunodeficiency virus SHIV(SF162P3). J Virol 75: 1990–1995. 11160699 10.1128/JVI.75.4.1990-1995.2001 PMC115146

[23] Parikh UM, Dobard C, Sharma S, Cong ME, Jia H, et al (2009) Complete protection from repeated vaginal simian-human immunodeficiency virus exposures in macaques by a topical gel containing tenofovir alone or with emtricitabine. J Virol 83: 10358–10365. 10.1128/JVI.01073-09 19656878 PMC2753130

[24] Henning T, Butler K, Mitchell J, Ellis S, Deyounks F, et al (2014) Development of a rectal sexually transmitted infection—HIV coinfection model utilizing Chlamydia trachomatis and SHIVSF162p3. J Med Primatol 43: 135–143. 10.1111/jmp.12103 24460742

[25] Patton DL, Cosgrove Sweeney YT, Rabe LK, Hillier SL (2002) Rectal applications of nonoxynol-9 cause tissue disruption in a monkey model. Sex Transm Dis 29: 581–587. 12370525 10.1097/00007435-200210000-00004

[26] Van Damme L, Ramjee G, Alary M, Vuylsteke B, Chandeying V, et al (2002) Effectiveness of COL-1492, a nonoxynol-9 vaginal gel, on HIV-1 transmission in female sex workers: a randomised controlled trial. Lancet 360: 971–977. 12383665 10.1016/s0140-6736(02)11079-8

[27] WHO (2014) Consolidated Guidelines on HIV Prevention, Diagnosis, Treatment and Care for Key Populations. Available: http://www.who.int/hiv/pub/guidelines/keypopulations/en/. Accessed 2015 Feb 10. 25996019

[28] Centers for Disease C, Prevention (2011) Interim guidance: preexposure prophylaxis for the prevention of HIV infection in men who have sex with men. MMWR Morb Mortal Wkly Rep 60: 65–68. 21270743

