# Supplementary figures and images for: Rectal Application of a Highly Osmolar Personal Lubricant in a Macaque Model Induces Acute Cytotoxicity but Does Not Increase Risk of SHIV Infection
Source: PLoS One. 2015 Apr 8;10(4):e0120021. doi: 10.1371/journal.pone.0120021 (PMC4390343; doi:10.1371/journal.pone.0120021)

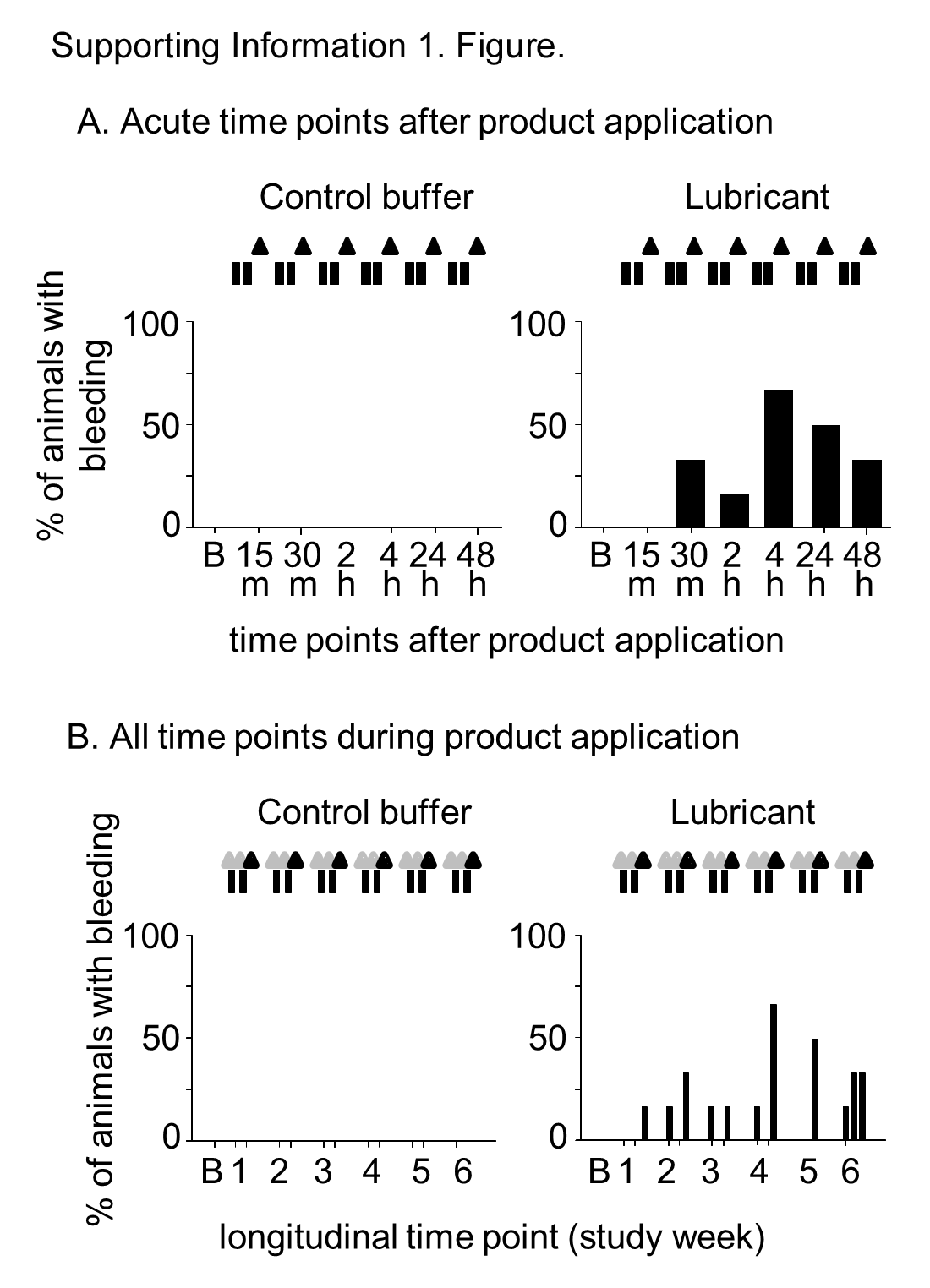

Supplement: S1 Fig — The percentage of macaques with visible blood in their rectal washes is plotted for time points acutely after product application (A), or for all time points of specimen collection (B). (TIF) [file pone.0120021.s002.tif]

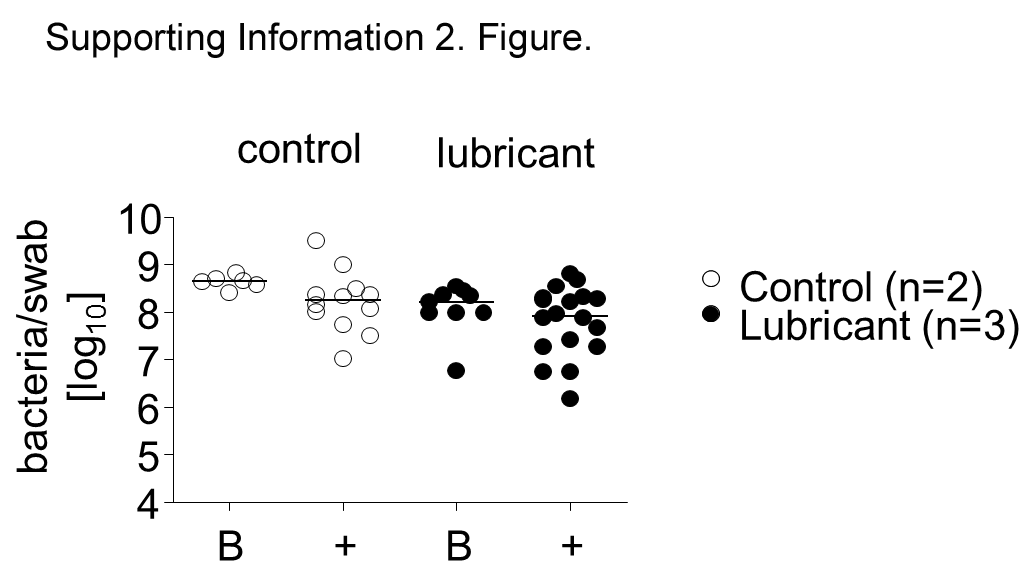

Supplement: S2 Fig — We found no significant difference (unpaired, two-tailed t-test) between the two arms; bacteria were cultured and counted from rectal swabs. “B” refers to baseline; three independent time points were analyzed. “+” refers to product-treated, six time points were analyzed. Data was combined from two controls, and three lubricant-treated animals. Lines are medians; open circles are from controls, filled circles from the lubricant group. (TIF) [file pone.0120021.s003.tif]

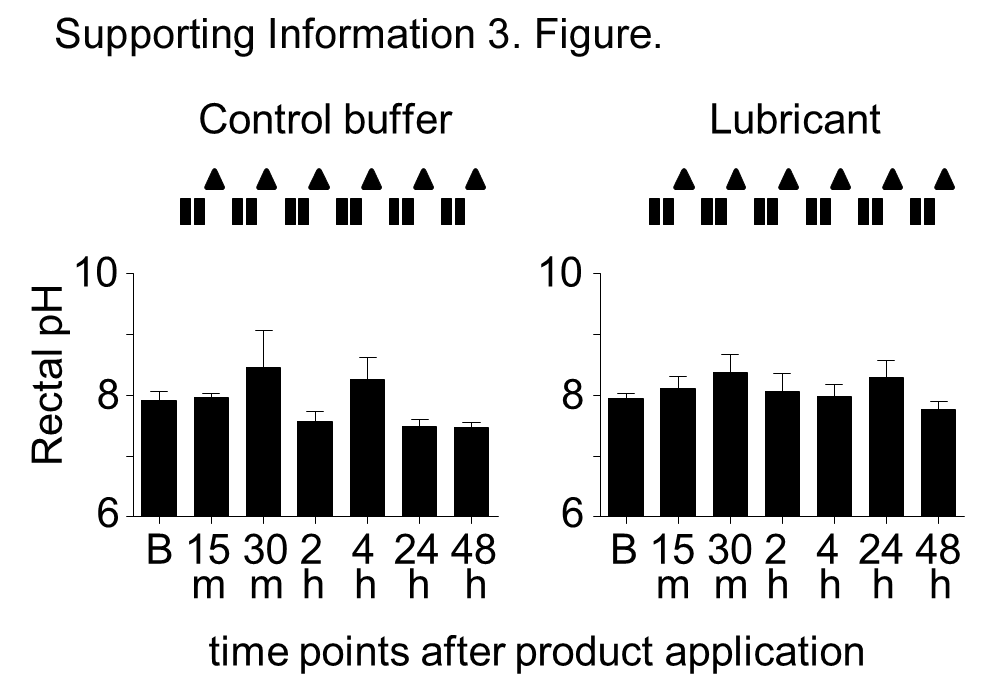

Supplement: S3 Fig — Application of a lubricant with pH of 4.4 does not significantly alter rectal pH. Rectal fluids were collected with a cotton swab, and immediately applied to pH strips. “B” baseline (Six independent time points); only “acute” time points (after second weekly product application) are shown; “m” minutes, “h” hours. Mean and SEM are shown; the schematic refers to study design shown in Fig. 1. (TIF) [file pone.0120021.s004.tif]

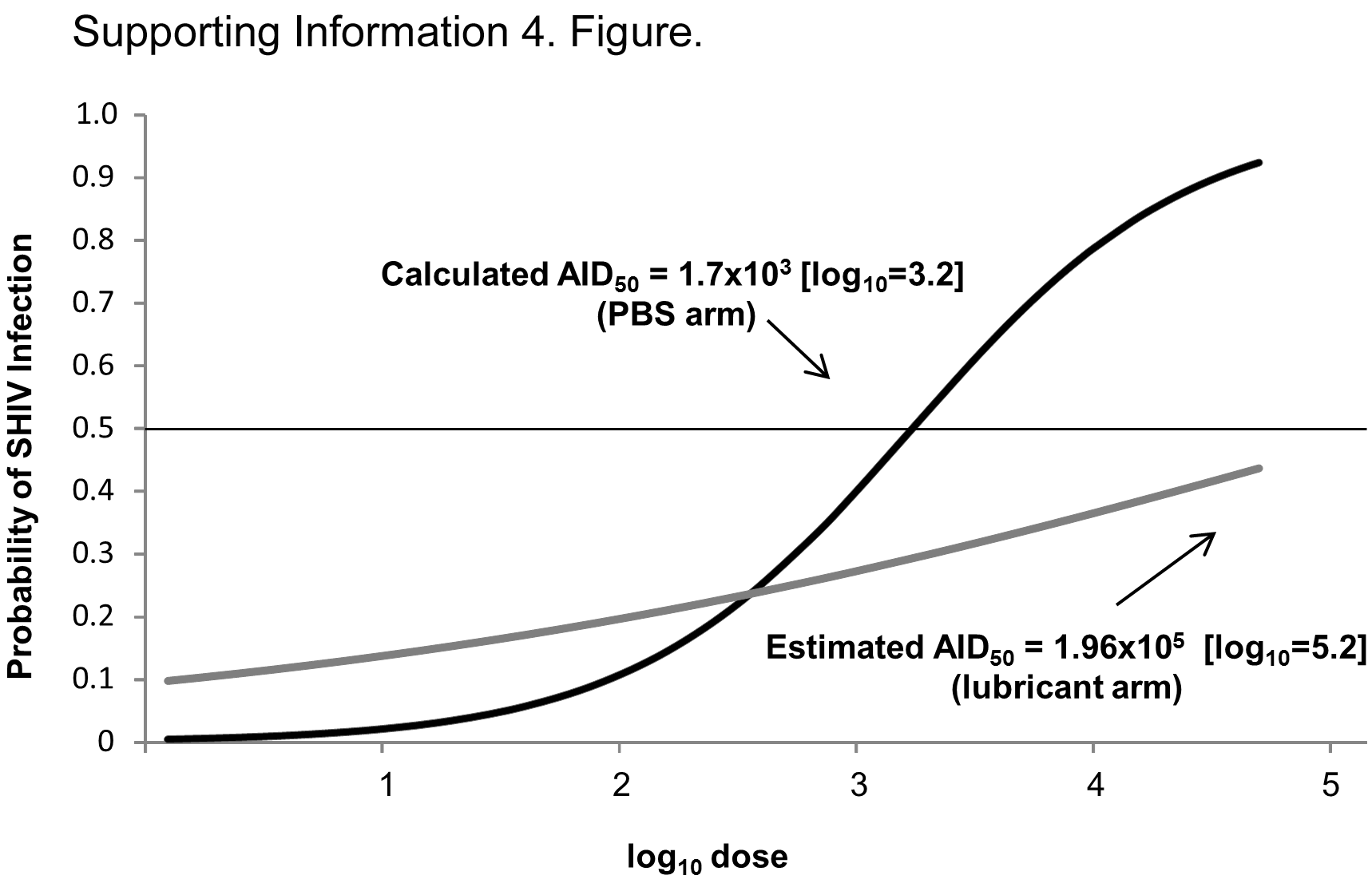

Supplement: S4 Fig — The estimated AID50 ratio (114) between the study arms did not show statistically significant difference (p = 0.45); shown here are the dose curves for the control buffer-treated (black line) and lubricant-exposed (grey line) animals; the log10 value for the lubricant arm was extrapolated. (TIF) [file pone.0120021.s005.tif]
