# Supplementary material for: Rectal Application of a Highly Osmolar Personal Lubricant in a Macaque Model Induces Acute Cytotoxicity but Does Not Increase Risk of SHIV Infection
Source: PLoS One. 2015 Apr 8;10(4):e0120021. doi: 10.1371/journal.pone.0120021 (PMC4390343; doi:10.1371/journal.pone.0120021)
Supplement: S1 Table — Sets1 and 2 were phosphate buffered saline (PBS)-treated controls (c); sets 3, 4, 5 and 6 were lubricant-treated (L); ‘+’ = animal infected; ‘−’ = animal not infected; historical controls are not shown here. The virus doses (in TCID50) are indicated in parenthesis next to every SHIV challenge. During the cytotoxicity testing phase of the study, animals 610214, 610268, and 607780 were PBS-treated, and animals CY9813, 609290, 609354, 603306, 609320, and 604962 were lubricant-treated. (PDF) [file pone.0120021.s006.pdf]

S1 Table. SHIV challenge design showing the animal IDs.

ID	set 1 (c)	set 2 (c)	set 3 (L)	set 4 (L)	set 5 (L)	set 6 (L)
**CY9813**	+ (25000)					
**72371**	+ (250)					
**82112**	+ (2500)					
**610214**	- (12.5)		- (250)	+ (1250)		
**610268**	- (12.5)		- (250)	+ (1250)		
**72187**	- (250)		- (1.25)	- (1250)	+ (2500)	
**81451**	- (250)		- (1.25)	+ (250)		
**81427**	- (2500)		+ (500)			
**90153**	- (1250)		- (50)	- (500)	- (1250)	- (5000)
**90398**	- (1250)		- (50)	- (500)	+ (500)	
**609290**		+ (5000)				
**609354**		+ (5000)				
**603306**		+ (2500)				
**609320**		+ (2500)				
**604962**					- (5000)	
**607780**		- (250)	- (1.25)	- (2500)	- (2500)	
**72203**		- (1250)			+ (5000)	
**81951**		- (1250)		- (500)	- (1250)	- (2500)
**81461**		- (500)	- (12.5)	- (250)	- (500)	- (25000)
**81559**		- (500)	- (12.5)	+ (250)		
**70782**						- (25000)

Sets1 and 2 were phosphate buffered saline (PBS)-treated controls (c); sets 3, 4, 5 and 6 were lubricant-treated (L); '+' =animal infected; '-' =animal not infected; historical controls are not shown here. The virus doses (in TCID50) are indicated in parenthesis next to every SHIV challenge. During the cytotoxicity testing phase of the study, animals 610214, 610268, and 607780 were PBS-treated, and animals CY9813, 609290, 609354, 603306, 609320, and 604962 were lubricant-treated.
